# Clinical outcomes after using patient specific instrumentation: is it worth the effort? A minimum 5-year retrospective review of 298 PSI knees

**DOI:** 10.1007/s00402-022-04593-0

**Published:** 2022-10-10

**Authors:** Rammohan R, Luke Nugent, Sarang Kasture, Muthu Ganapathi

**Affiliations:** 1 Ysbyty Gwynedd, Betsi Cadwaladr University Health Board, Bangor, LL572PW Wales; 2 Present Address: Royal Shrewsbury Hospital, The Shrewsbury and Telford Hospital NHS Trust, Shrewsbury, SY3 8XQ England; 3 Present Address: Stepping Hill Hospital, Stockport NHS trust, Stockport, SK27JE England

**Keywords:** Knee osteoarthritis, Total knee arthroplasty, Patient specific instrumentation, Magnetic resonance imaging, Patient reported outcome measures, Retrospective study

## Abstract

**Introduction:**

Use of patient specific instrumentation (PSI) for performing total knee arthroplasty (TKA) has been shown to improve component positioning but there is dearth of evidence regarding clinical outcomes. The aim of our study was to report patient satisfaction and functional outcome scores of patients who underwent PSI TKAs at minimum 5 year follow up.

**Methods:**

This is a retrospective study of a prospectively collected data of patients who underwent PSI TKAs between January 2012 and October 2015 under a single surgeon. Patient Reported Outcome Measures (PROMs), patient satisfaction questionnaires, surgeon directed 3D planning changes and intra-operative changes were collected and analysed.

**Results:**

The cohort included 298 consecutive PSI TKAs performed on 249 patients at a mean age of 71 years (range: 49–93 years). On an average 4 changes were made for each knee during 3D planning compared to preliminary plan. Intra-operative implant size change was required only in 3% (10 knees). The PROM scores were collected at a mean follow-up period of 6.8 years (range: 5.0–8.6 years) for 224 knees. Oxford Knee Score improved from median pre-operative score of 18 (IQR: 13–24) to median post-operative score of 44 (IQR: 40–47) with a median gain of 23 (IQR: 16–30). The median modified Forgotten Joint Score was 87.5 (IQR: 54.4–98.1). For the Beverland questionnaire, 75% (*n* = 166) reported being “Very Happy” and only 4% (*n* = 9/222) were ‘Never Happy’.

**Conclusion:**

Excellent patient satisfaction and functional scores at mid-term can be achieve d using PSI technique to perform TKA with careful surgeon directed pre-operative planning.

## Introduction

Total knee arthroplasty (TKA) is a definitive surgical treatment for end-stage knee osteoarthritis which can reliably relieve pain and correct deformity [1]. Developments in implant design and surgical techniques have led to improved survival of the implant with modern implants lasting up to 25 years [2]. Despite improved longevity, 20% of patients continue to report pain and functional limitation after TKA [3]. Restoration of the alignment and adequate soft tissue balancing are established factors that affect outcomes after TKA [4, 5]. Patient specific instrumentation (PSI) was developed to improve the component alignment, accuracy, and precision in comparison to the conventional technique of TKA, whilst also improving theatre efficiency [6]. Improved accuracy and alignment were also postulated to improve patient satisfaction and functional outcome. Studies have confirmed this predicted improvement in component alignment [6–8], however, patient satisfaction outcomes have been variable or at best non-inferior to TKA using conventional instrumentation [9]. This study aimed to evaluate functional outcomes and satisfaction of patients who underwent TKA using MRI based PSI at mid-term with a minimum of 5 years follow-up.

## Methodology

We present a retrospective review of a prospectively maintained database. Our cohort of patients underwent TKA procedures performed using Magnetic Resonance Imaging (MRI) based PSI technique at a single institution, performed by the senior author or under his direct supervision between January 2012 and October 2015. There were no other exclusion criteria apart from the contra-indications to undergo an MRI scan or when the MRI scan was not possible due to organisational issues. The patient demographics, American Society of Anaesthesiologists (ASA) grade, Body Mass Index (BMI), pre-operative surgical 3-D planning details, intra-operative clinical assessment of stiffness, intra-operative changes to the approved plan, additional procedures, revisions, and Patient Reported Outcome Measures (PROM) were collected for the patients included in the cohort. The PROM scores included pre-operative and post-operative Oxford Knee Score (OKS) [10], Modified Forgotten Joint Score (MFJS) [11], 0–10 point scales for pain and stability, and Beverland questionnaire on patient satisfaction [12]. Patients’ co-morbidities at the latest assessment were stratified according to the modified Charnley class [13]. PROM and patient satisfaction data at mid-term were obtained initially at face-to-face interviews in the out-patient clinics. However, subsequently due to the COVID-19 pandemic, the majority of the interviews were conducted via telephone as part of virtual review clinics. Post-operative OKS scores obtained at one-year face to face follow-up clinics were also available for 98 of the first 100 knees of this cohort (one patient had died and one knee had been revised).

Statistical Analysis was performed using R software (version 4.0.0, R Foundation for Statistical Computing, Vienna, Austria). The continuous data were presented as mean, range and Standard Deviation (SD) and the quantitative data which were not normally distributed were presented as Median and Interquartile range (IQR). Comparative analyses were performed using independent t-tests for parametric data, the Wilcoxon-rank sum test was used for paired, non-parametric, ordinal outcome measures. Statistical significance was defined as *p* < 0.05.

### Planning for PSI

**Fig. 1:**
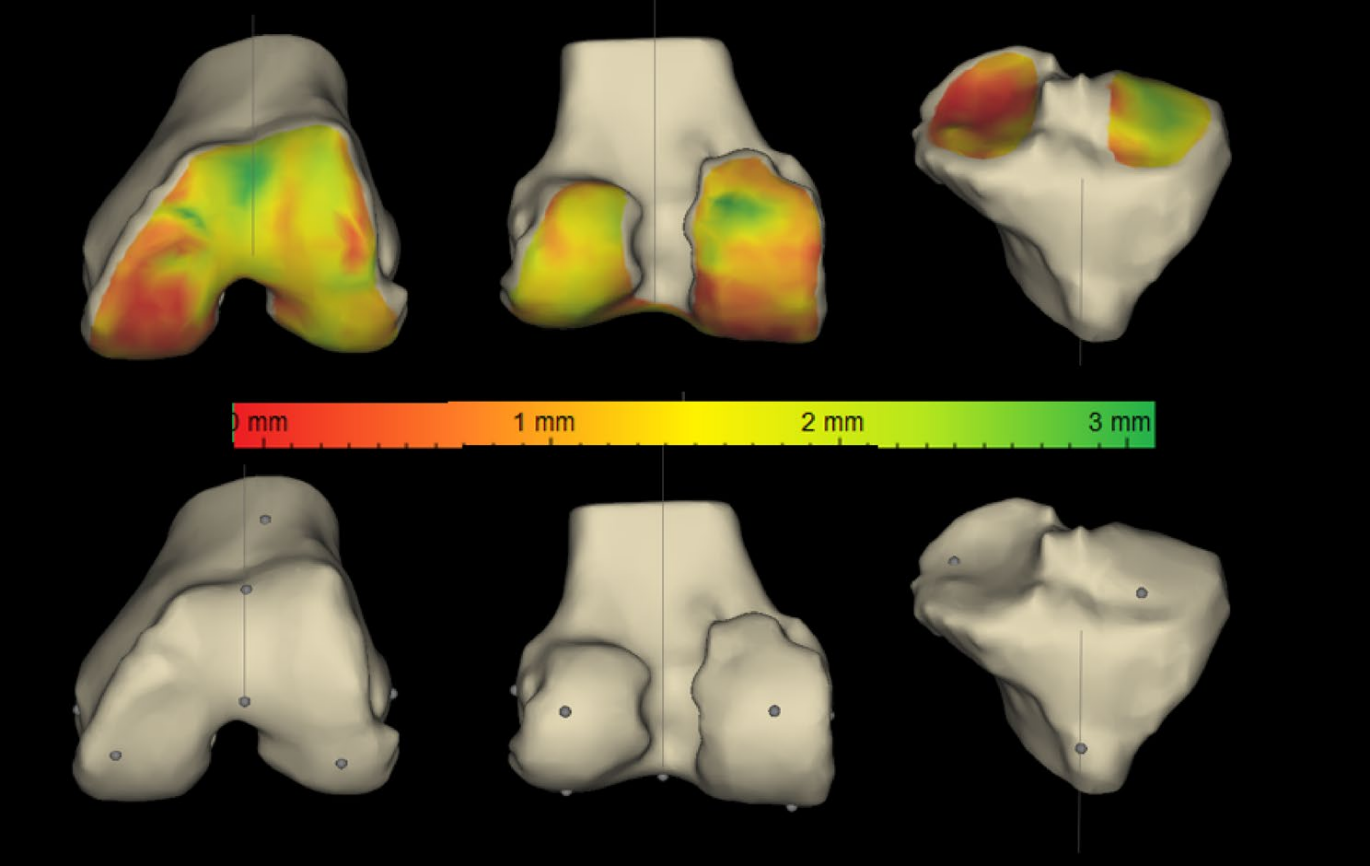
Cartilage mapping denoting cartilage loss at the resection reference points

MRI scan of the ipsilateral hip, knee and ankle was performed, and the images were sent to Materialise^®^ (Leuven, Belgium) for planning and design of patient-specific 3D moulds. The default pre-operative parameters for bone resection and implant alignment were set by the senior author as shown in Table 1. A preliminary plan was created by the technicians based on these parameters. This plan was carefully modified by the senior author before approval, which was then used to create femoral and tibial 3D moulds (pin positioning guides). The cartilage mapping unique to the ZPSIP^®^ planning software helped assess the loss of cartilage at the resection reference points (Fig. 1). Both distal femoral and proximal tibial resections were planned to be perpendicular to the respective mechanical axes with aim of achieving neutral limb alignment.

**Table 1:** Default pre-operative parameters for bone resection and component alignment

Parameter	Default setting
Distal femoral resection angle	Neutral (0 degrees to the mechanical axis)
Flexion extension angle of the femoral component	3 degrees
Distal femoral resection	10 mm
Posterior femoral resection	11.5 mm (NexGen CR Flex)/10 mm (Persona CR)
Femoral rotation	Trans-epicondylar axis
Referencing	Posterior
Proximal tibial resection angle	Neutral (0 degrees to the mechanical axis)
Tibial slope	7 degrees (NexGen CR Flex)/5 degrees (Persona)
Proximal tibial resection	10 mm
Tibial rotation	0 degrees (to the anatomical axis)

**Fig. 2:**
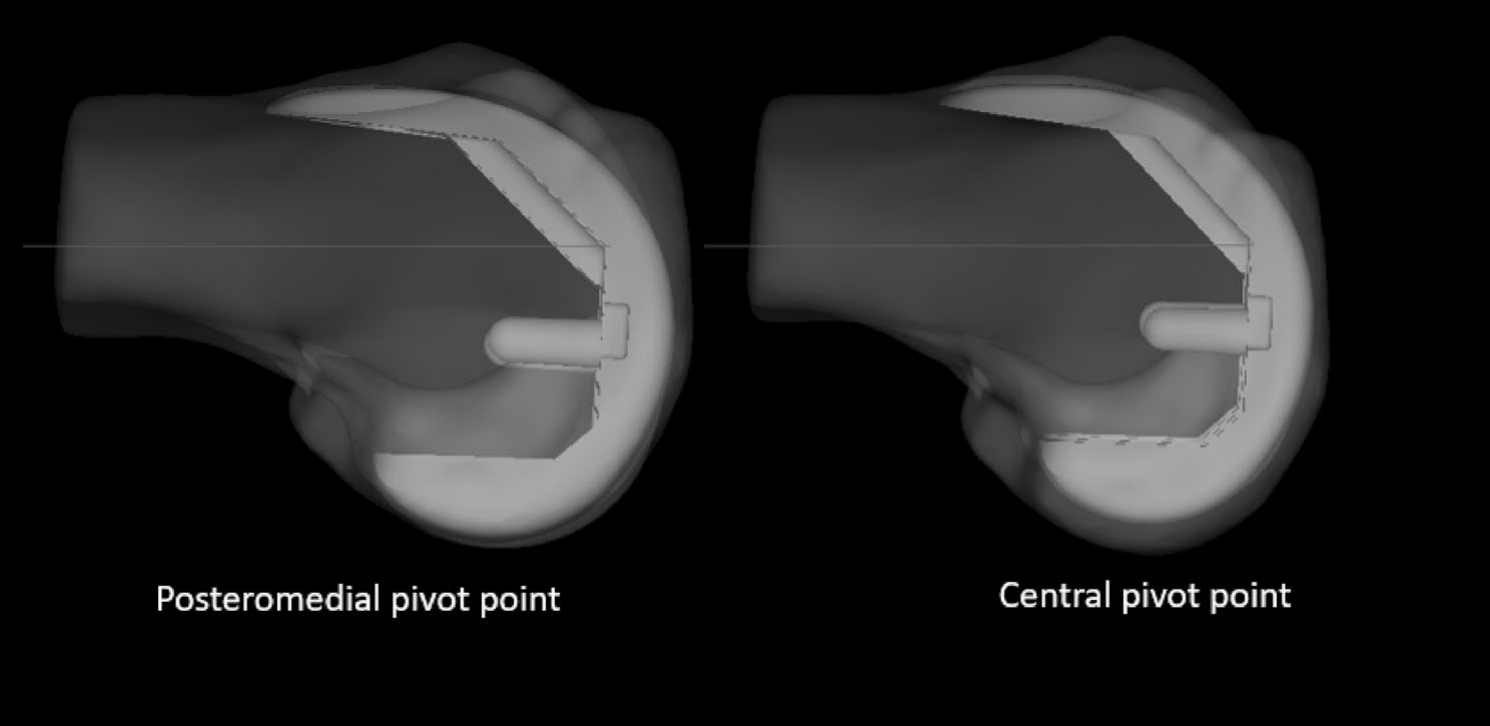
Rotational pivot—Implant superimposed on the native femur showing the rotational pivot point on posteromedial condyle resection (4 ER relative to PCA). Implant superimposed on the native femur showing the of rotational pivot point on posteromedial condyle resection (4° ER relative to PCA). The posterior condylar offset is preserved with posteromedial pivot point unlike when the pivot point is at the centre of the distal femur which results in loss of posterior condylar offset.

One of the fundamental principles followed was to resurface the medial femoral condyle both distally and posteriorly to closely match implant thickness. This was made possible by the ZPSIP^®^ planning software (Materialise^®^ and Zimmer-Biomet^®^), which has the rotational pivot point (when setting up the rotation of the femoral component) at the posterior aspect of the medial femoral condyle when a posterior referencing option is chosen. This allowed measured resection of the posterior medial condyle close to implant thickness regardless of the degree of external rotation dialled in relative to the posterior condylar axis (PCA). This is different from conventional instrumentation where the rotational pivot is at the centre of the distal femur resulting in greater resection of the posterior medial condyle than the implant thickness with any external rotation (Fig. 2).

**Fig. 3:**
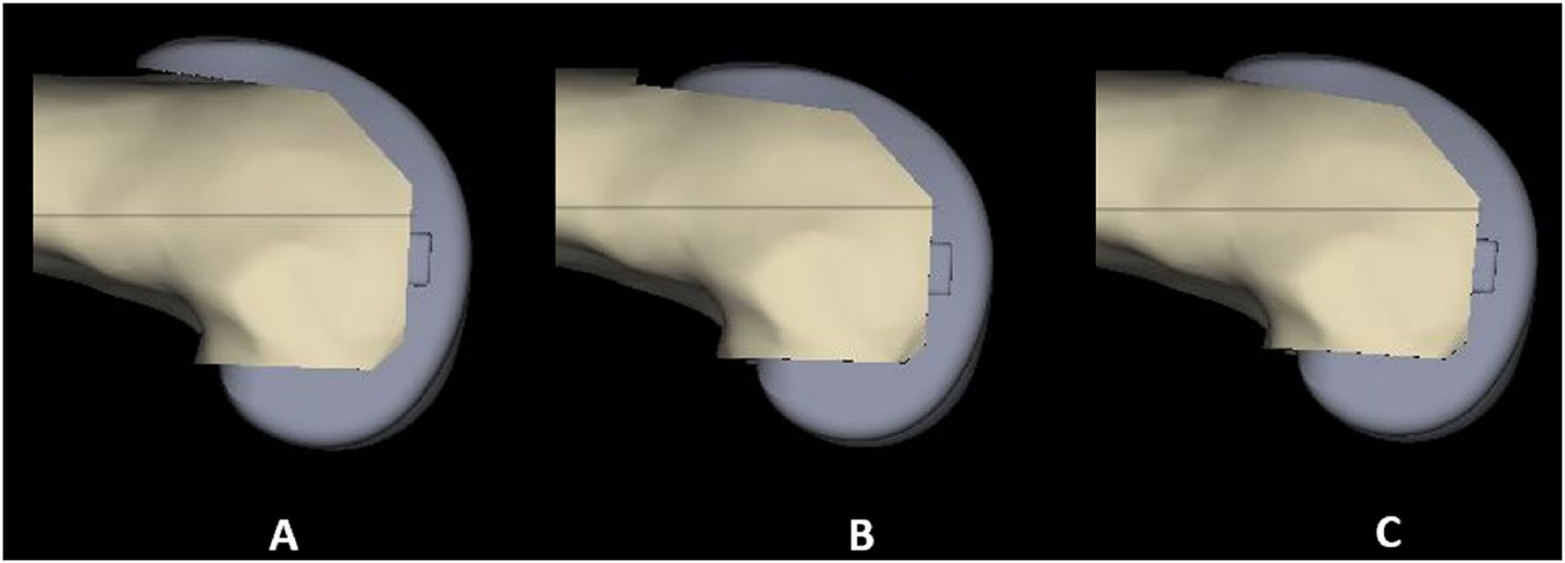
Femoral component alignment. A Default femoral component chosen by the software. B Notching is obvious if one size less is chosen. C. Minor adjustment by 2° flexion, 1 mm anterior shift and 1 mm proximal shift allowed one size less to be selected without significant notching

We also aimed to avoid over-stuffing the patellofemoral joint. With the posterior referencing system, the software often suggested the larger size femoral component to avoid notching if the actual size was in between two sizes. In such cases, we opted for a smaller size to avoid overstuffing the patellofemoral compartment. To minimise notching, if required, the femoral component was flexed to a maximum of further 2 degrees and shifted 1 mm anteriorly (Fig. 3). A corresponding proximal shift of the distal femur cut was also planned to maintain the proportional change in the medial femoral joint line.

**Fig. 4:**
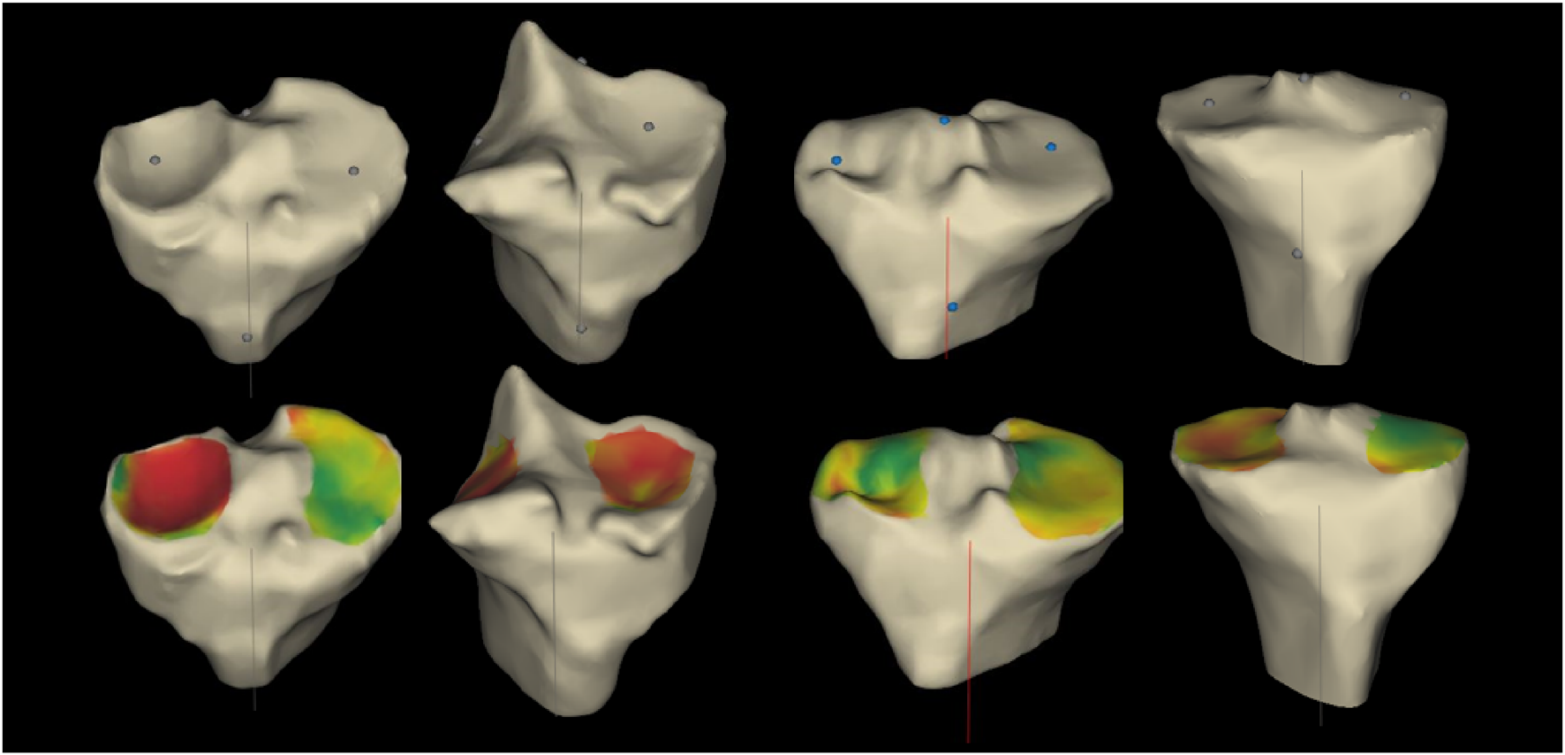
Tibial resection planning. Tibial resection reference points and cartilage wear at the reference points were taken into account when planning tibial resection

On the tibial side, while a 10 mm resection was aimed for, the final adjustment was done depending on where the actual resection reference point was in relation to the cartilage wear as well as the coronal plane deformity with conservative resection planned in knees with valgus deformity (Fig. 4). The default tibial rotation was based on the Cobb’s method [14]. We found this to be somewhat internally rotated compared with the junction of the medial third and lateral two-thirds of the tibial tuberosity. Hence in most cases, some external rotation to the tibial component was added at the final planning stage. Careful analysis of the 3D model of the proximal tibia allowed for an appreciation of the extent of osteophyte formation and optimal implant sizing. A small postero-lateral overhang was accepted to avoid under-sizing the tibial component.

### Surgical technique

The medial parapatellar approach without a tourniquet was used in all cases. The distal femoral cut and the 4-in-1 bone cuts were done using conventional cutting blocks based on the pins drilled through the holes of the PSI guides. If there was a large flexion deformity such that the planned resection thickness would be inadequate (based on clinical judgement), the distal cutting guide was moved to the plus 2 position for the initial cut. Before the 4-in-1 cuts were made, if more than minimal notching was felt to be present (up to 2 mm notching was accepted), the femoral component was up-sized. The posterior cut was never changed. Holes for pins for placement of the proximal tibial resection guide were drilled through the tibial PSI guide. Proximal tibial resection was done through the conventional cutting blocks. The cutting guide was moved to a + 2 position if the resection appeared inadequate.

**Fig. 5:**
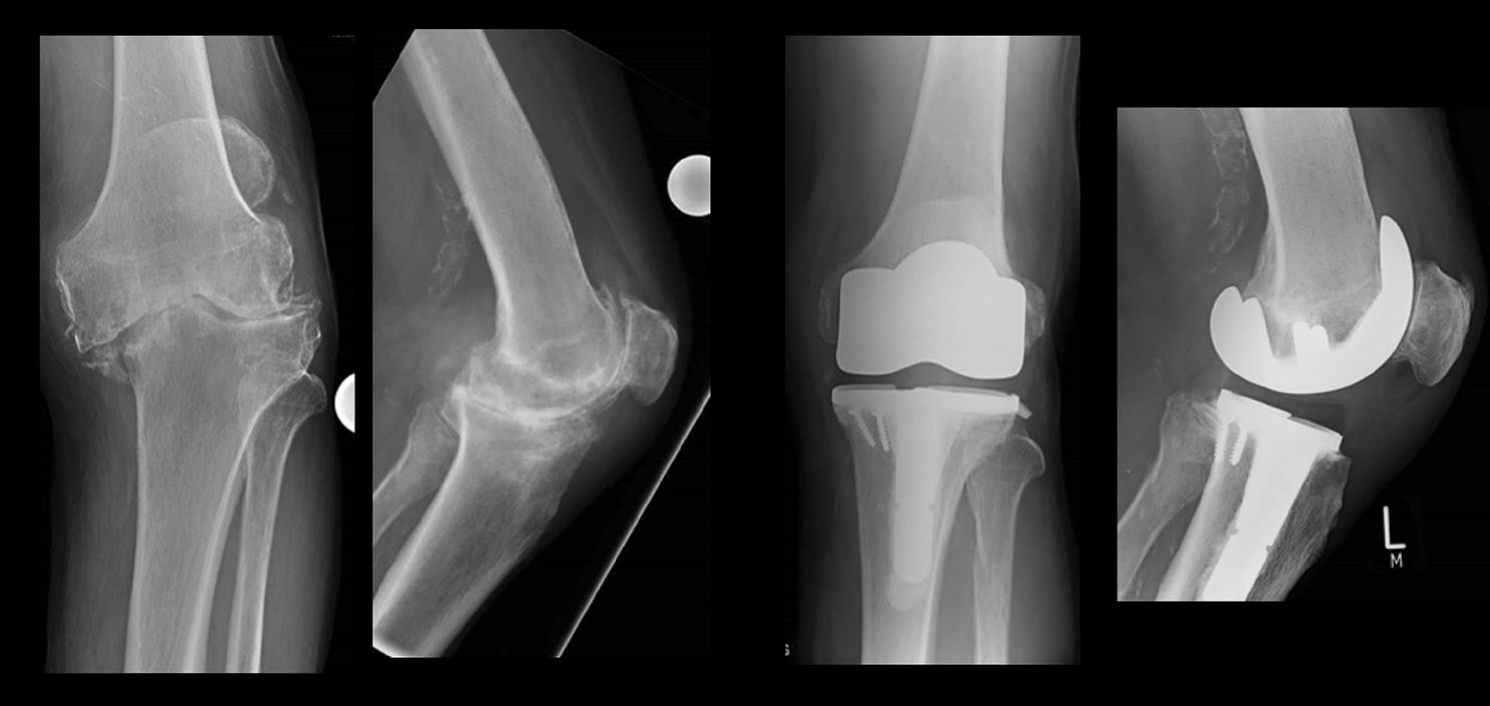
OA with severe varus deformity (7 changes done to preliminary plan before approval)

**Fig. 6:**
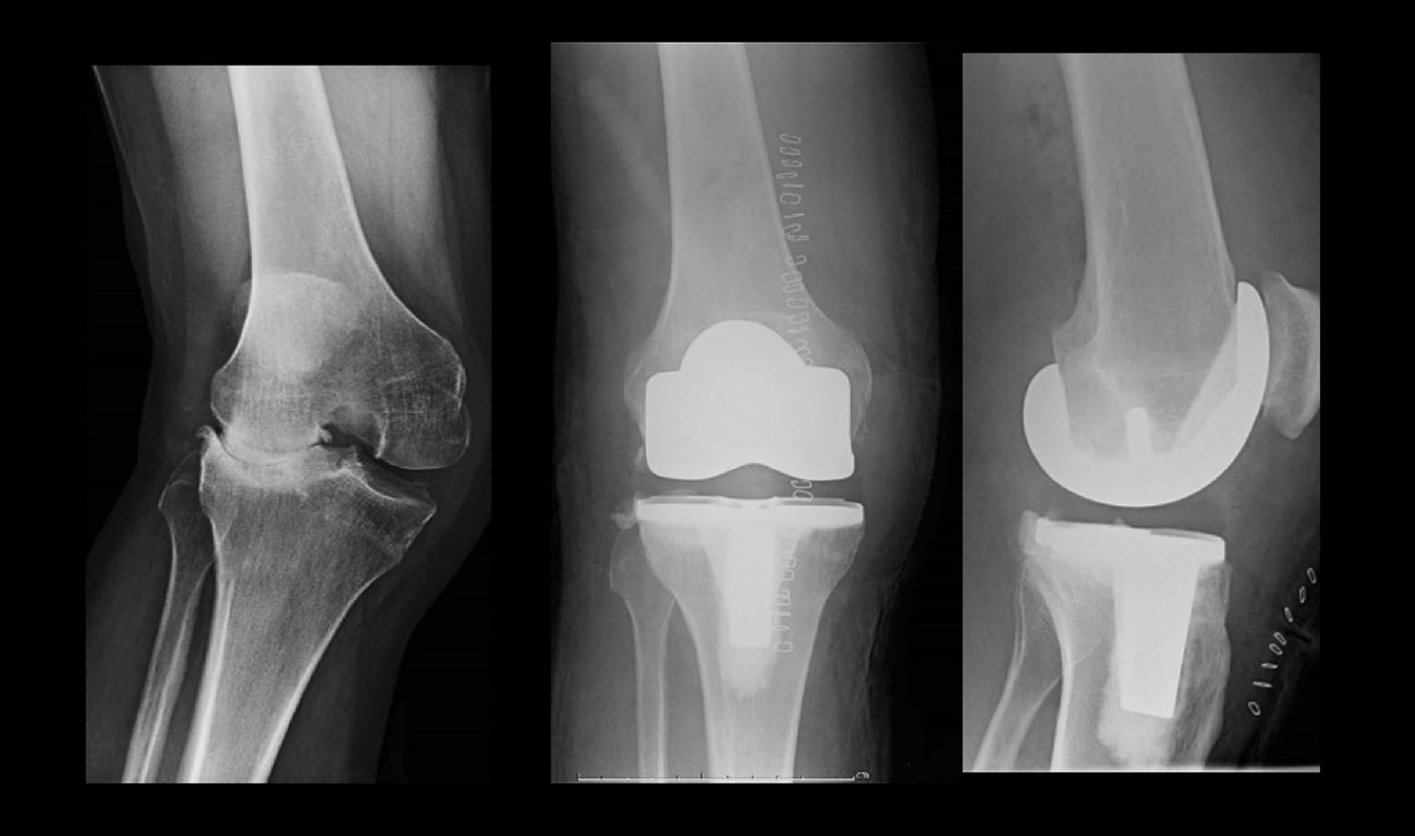
OA with severe valgus deformity (7 changes done to preliminary plan before approval)

A 10 mm tibial insert was aimed for. Slight lateral laxity was accepted but efforts were made to achieve medial stability both in extension and flexion. Any osteophytes were removed. Any further distal femoral or proximal tibial resections in 2 mm increments were done as required to achieve adequate extension and flexion gaps. Soft tissue release was also done as required. Medial soft tissue release often was done by inside out needle “pie-crusting” of the medial collateral ligament [15], but in some cases, more extensive releases including posteromedial release and partial posterior cruciate ligament (PCL) release were also required. The lateral release included postero-lateral corner capsular release and pie-crusting of the iliotibial band. Primary patellar resurfacing was not done in any patient, but circumferential resection of osteophytes, electrocautery denervation [16] and patelloplasty [17] were routinely done. In all but one knee, cruciate retaining (CR) femoral components were used regardless of the degree of coronal deformity, stiffness, or flexion deformity (Fig. 5 and Fig. 6). ‘POLO test’ (Pull Out, Lift Off) was routinely used to check adequate soft tissue balancing [18]. Once the trial reduction was satisfactory definitive components were implanted. The wound was closed in layers without a drain.

## Results

The evaluated cohort consisted of 298 consecutive TKAs performed on 249 patients (Table 2), with 142 women (170 knees) and 107 men (128 knees). Both knees were replaced in 49 patients (nine patients underwent bilateral replacements at the same sitting). The mean age at the time of surgery was 71 years (range 49–93). A NexGen CR-Flex Fixed Bearing implant (Zimmer-Biomet, Warsaw, USA) was used in most cases (91%, *n* = 272) of which 49 were Gender type. Persona CR implants (Zimmer-Biomet, Warsaw. USA)) were used in 25 cases. Most components were cemented (78.2%, *n* = 233), with 21.8% (*n* = 65) being uncemented implants. The mean BMI was 32.0 (Standard Deviation [SD] = 5.7, range 19–48.2) with 28.81% (*n* = 83) having BMI 35 or more and 11.07% (*n* = 33) having a BMI of 40 or more. More than a quarter of the knees had fixed flexion deformity between 15 and 25 degrees (26.83%, *n* = 80) and 9% (*n* = 27) of the knees were stiff with flexion less than 100 degrees. While a majority at mid-term review belonged to modified Charnley Class B (54.5%, *n* = 122), one third belonged to Charnley class C (Table 2).

**Table 2:** Patient demographics

	*N* (percentage)	Mean ± SD (Range)
Age, years		71 ± 8.8 (49–93)
BMI		32.0 ± 5.7 (19–48.2)
Sex		
Male	128 (43%)	
Female	170 (57%)	
ASA		
1	17 (5.7%)	
2	219 (73.5%)	
3	61 (20.5%)	
4	1 (0.3%)	
Type of implant		
Cemented	233 (78.2%)	
Uncemented	65 (21.8%)	
Modified Charnley class (at mid-term review)		
A (Unilateral TKA)	26 (11.6%)	
B (Bilateral TKA or unilateral TKA with contralateral knee arthritis)	122 (54.5%)	
C (TKA with remote arthritis and/or medical condition that affected their ability to ambulate)	76 (33.9%)	

Thirty-two patients with 40 TKAs has died between 5 and 100 months after the Index procedure at the time of mid-term review and none of them had undergone revision. Five patients underwent revision during the study period and their functional outcome scores were therefore not collected. 13 patients could not be contacted. A further 16 patients were unable to respond because of medical reasons (Of the 10 who were residents of assisted care home or nursing home, 3 had stroke, 4 had dementia, 2 had recent lower limb fractures, 1 had malignancy; 6 were unable to answer the questions because of other medical problems including severe dyspnoea). PROM scores and patient satisfaction responses were collected in all other patients (*n* = 224, 75%) at mid-term with a mean follow-up period of 6.8 years (SD = 0.9, range: 5.0–8.6 years). Two patients underwent subsequent patellar resurfacing during the study period, but their functional scores were included.

Analysis of planning changes: the frequency of changes to the 3D plan before final approval by the senior author is shown in Table 3. This excludes changes in the tibial rotation and resultant antero-posterior and mediolateral shifts (as these were not readily quantifiable). Regardless, an average of almost 4 changes were done for each case before final approval. The femoral component was down-sized on 138 occasions and up-sized on 4 occasions. The tibial component was down-sized on 46 occasions and up-sized on 66 occasions. In the final approved plan, minimal notching of the anterior femoral cortex was observed and accepted in 193 patients (65%) to avoid over-stuffing the patello-femoral compartment. A minimal postero-lateral overhang of the implant was accepted in 87 cases (30%) to avoid under-sizing of the tibial component.

**Table 3:** Changes to default plan and warnings accepted by Senior author during pre-operative planning (*n* = 298)

	Number of changes (percentage)
Femur	
Flexion–extension angle	146 (49%)
Distal resection depth	142 (47%)
Posterior resection depth	102 (34%)
Notch warning rejected	151 (51%)
Default femur size changed	142 (48%)
Tibia	
Proximal resection depth	80 (27%)
Posterior slope	0 (0%)
Posterolateral overhang warning rejected	192 (64%)
Default tibial size changed	112 (38%)
Total number of changes	1067 (approximate: 4 changes per knee)

Analysis of intra-operative changes: PSI guide fit was considered adequate in all cases. Intra-operative changes to the distal femoral resection (plus 2 mm or more than pre-planned resection) were necessary in 43 cases (14%). Similarly, a plus 2 mm or more of proximal tibial resection was required in 42 cases (14%). Pre-planned implants were changed in only 1.3% of the femoral components (up-sized in 1, down-sized in 2 and changed to Gender type in 1) and 2% of the tibial components (up-sized in 5 and down-sized in 1).

Medial releases were performed in 46% of cases (*n* = 137/298) and lateral releases in 13.8% (*n* = 41/298). No releases were required for 40% of cases (*n* = 119/298). To improve patella tracking, limited lateral parapatellar release was done in 5.4% (*n* = 16/298) of cases. 10 mm inserts were used in 62.75% (187/298) cases. The remainder consisted of 9 mm, 11 mm, 12 mm and 14 mm inserts, used in 1.34% (4/298), 1.67% (5/298), 29.53% (88/298) and 4.69% (14/298), respectively.

**Fig. 7:**
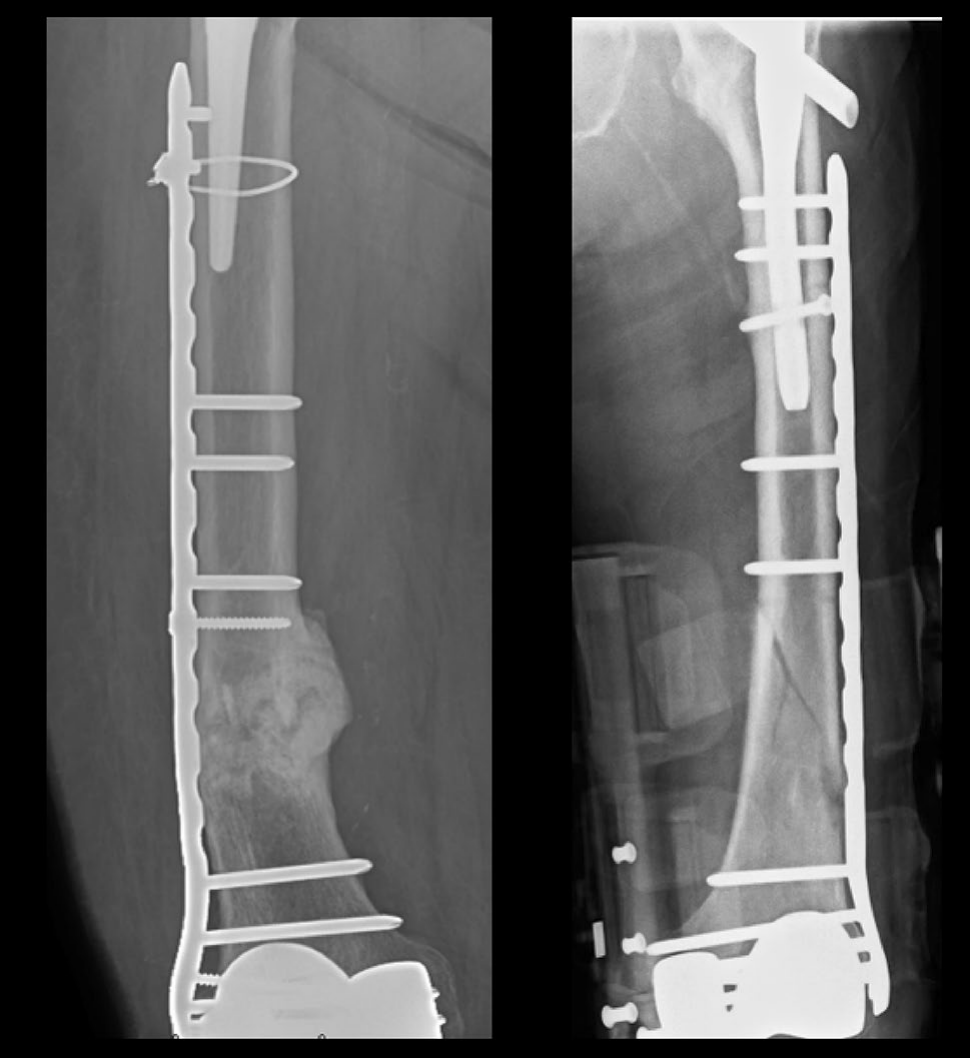
Post-operative radiographs of two patients who had peri-prosthetic fractures proximal to knee prosthesis, without the loosening of the prosthesis, managed by open reduction and internal fixation

Five knees had been revised at the time of mid-term review. Two knees required manipulation under anaesthetic to improve range of movement at 1- and 4-months post-surgery. One patient had successful arthroscopic exploration and soft tissue debridement to relieve catching sensation at the lateral aspect. Secondary patellar resurfacing had been done in three knees (pain improved = 1; no change in pain = 1; pain worsened = 1). There were no deep infections. There were two Vancouver type C peri-prosthetic fractures which were away from the femoral component including one through an osteolytic myelomatous lesion (Fig. 7).

The primary measures of outcome at mid-term are described in Table 4, which lists the OKS, MFJS, pain score, stability score and Beverland questionnaire on patient satisfaction. The median post-operative OKS at mid-term was 44 (IQR: 40–47) with a median gain of 23 (IQR: 16–30). The median OKS of the first 100 patients at 1-year follow-up was 43 (IQR: 36.5–45.5) and the median gain was 23 (IQR: 16.5–28). There was no statistically significant difference between the median post-operative OKS scores (Wilcox-rank-sum test, *p*-value = 0.64) or the median gain in the OKS (Wilcox-rank-sum test, *p*-value = 0.64) for the two sets of scores collected at one-year post-operative and at mid-term.

**Table 4:** PROM scores and patient satisfaction at the mid-term review

Parameter (number)	Median pre-operative score (IQR)	Median post-operative score (IQR)	Median post-operative gain (IQR)
Oxford knee score	18 (13–24)	44 (40–47)	23 (16–30)
Modified forgotten joint score	–	87.5 (54.4–98.1)	–
Pain score	–	0 (0–2)	–
Stability score	–	0 (0–0)	–

The MFJS tests the patient’s awareness of the implant’s presence, with higher scores indicating lesser perception of the prosthetic joint. This MFJS is calculated using a questionnaire containing 10 questions with each response scored between 0 and 4 and converted to a percentage (range 0–100). A score of 100 refers to no perception of the prosthetic joint and a score of 0 refers to complete awareness of the prosthetic joint. The median MFJS in our study was 87.5 (IQR 51.9–97.5) with a 51% (*n* = 114/224) reporting score of 87.5 or more.

**Fig. 8:**
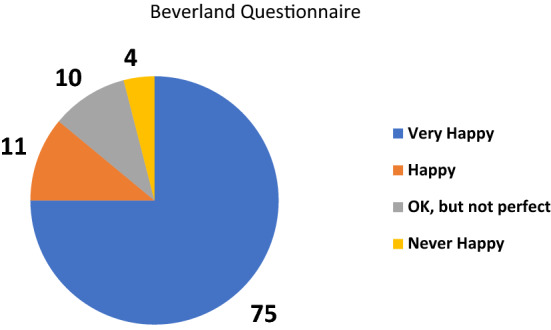
Beverland questionnaire response on satisfaction (In percentage, *n* = 222 knees)

Pain was measured using a 0–10 points scale, with 0 representing ‘no pain’ and 10 representing ‘worst pain’. 78% (*n* = 173/222) reported a pain score of 2 or less. Patients were also asked about their subjective feeling of stability (how secure they felt their knee was) using a 0–10 points scale, with 0 representing ‘fully secure knee’ and 10 representing ‘insecure knee’ that required guarding even with the activities of daily living. However, the question was not designed to differentiate the cause (e.g., coronal/sagittal instability, patellofemoral problems, muscle weakness, adjacent joint problems, and neurological issues). 87% (*n* = 193/221) had scores of 2 or less implying that they felt their knees were very secure. With regards to patient satisfaction, using the Beverland questionnaire 75% (*n* = 166/222) responded that they were “Very Happy” and only 4% (*n* = 9/222) reported that they were ‘Never Happy’ (Fig. 8).

**Fig. 9:**
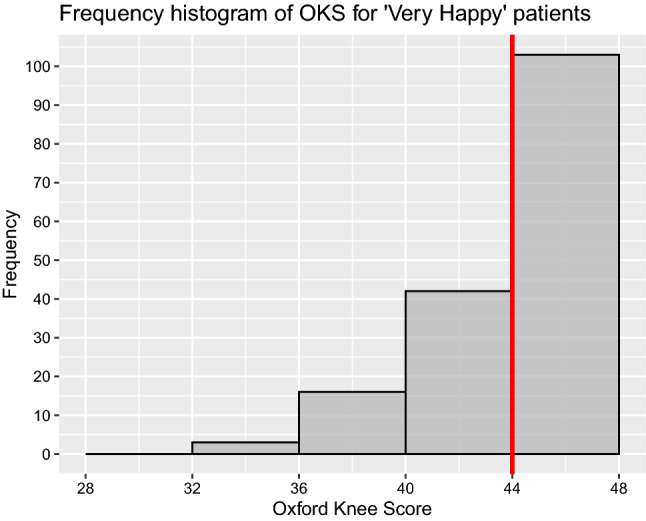
Histogram of OKS scores of patients who responded ‘Very Happy’ for Beverland questionnaire. The red coloured line represents the median

**Fig. 10:**
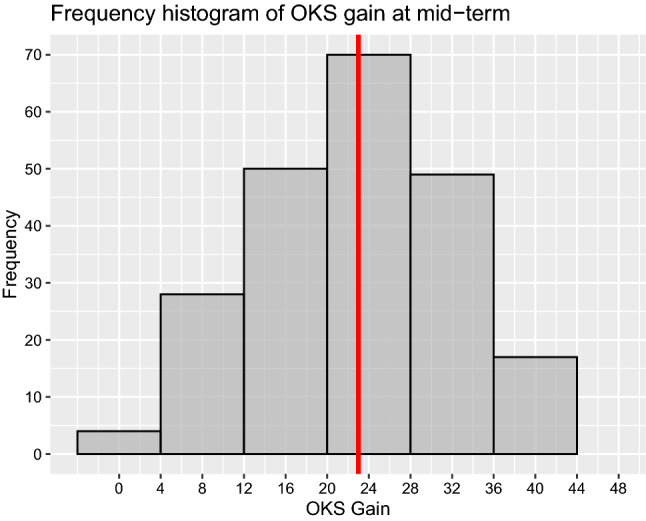
Histogram of OKS gain of patients who responded ‘Very Happy’ for Beverland questionnaire. The red coloured line represents the median

**Fig. 11:**
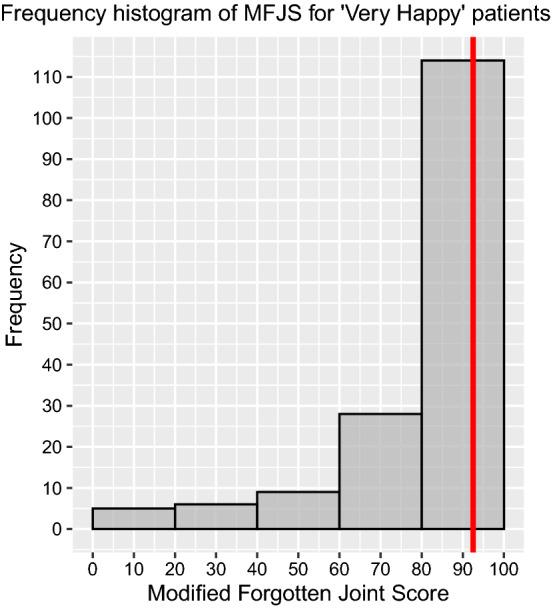
Histogram of MFJS scores of patients who responded ‘Very Happy’ for Beverland questionnaire. The red coloured line represents the median

Subgroup analysis of post-operative OKS (Table 5) showed significantly better scores in males and Charnley Class A/B groups compared to females and Charnley Class C group. There was no statistically significant difference in the other subgroup analysis (age, ASA grades, BMI and cemented/cementless) with regards to post-operative OKS. Subgroup analysis of Beverland questionnaire responses showed a statistically significant difference between the groups in relation to gender, BMI, ASA and Charnley functional status (Table 6). Males with BMI less than 35.0, ASA grades 1/2 and Charnley functional class A/B were more satisfied. On further analysis of ‘Very Happy’ (*n* = 166) patients on the Beverland questionnaire, the median post-operative OKS, gain in OKS and MFJS were 45 (IQR 43–47), 25 (IQR 19–31) and 92.5 (IQR 77.5–100), respectively (Figs. 9, 10, 11). For the same sub-group, the median pain score and stability scores were 0 (IQR 0–1) and 0 (IQR 0–0), respectively.

**Table 5:** Subgroup analysis of primary outcome measure

Group	Categories	Number of knees	Median pre-op OKS (IQR)	Median post-op OKS (IQR)	Student *t*-test *p*-value
Sex	Male	95	19 (13–28)	45 (41–47)	0.012
	Female	129	18 (13–23)	44 (38–46)	
Age	Age < = 70 years	109	18 (13–24)	44.5 (39–47)	0.494
	Age > 70 years	115	18 (13–25)	44 (40–46)	
BMI	BMI < = 35.0	168	20 (15–25)	44 (40–46)	0.734
	BMI > 35.0	56	14 (9.5–19.5)	45 (40–47)	
ASA	ASA 1 and 2	183	19 (13–25)	45 (40–47)	0.206
	ASA 3 and 4	41	16 (13–20)	43.5 (38–46)	
Implant	Cemented	177	19 (13–25)	45 (40–47)	0.562
	Uncemented	47	16 (13–21.5)	44 (40.5–46)	
Charnley class	Class A and B	148	20 (14–25)	45 (41–47)	< 0.001
	Class C	76	16 (12–20)	43 (33.5–46)

**Table 6:** Subgroup analysis for Beverland questionnaire responses (Chi-square test with Yate's correction)

Group	Categories	Number of knees	Very happy	Happy	OK, but not perfect	Never happy	Chi-square test with Yate’s correction: *p*-value
Sex	Male	95	75	13	3	2	0.015
	Female	129	91	12	19	7	
Age	Age < 70 years	109	78	17	10	2	0.089
	Age > 70 years	115	88	8	12	7	
BMI	BMI < = 35.0	168	128	15	19	5	0.080
	BMI > 35.0	56	38	10	3	4	
ASA	ASA 1 and 2	183	142	15	18	6	0.013
	ASA 3 and 4	41	24	10	4	3	
Implant	Cemented	177	127	22	19	7	0.466
	Uncemented	47	39	3	3	2	
Charnley class	Class A and B	148	121	12	10	4	0.004
	Class C	76	45	13	12	5

## Discussion

Patient satisfaction and functional scores are now increasingly being used to evaluate the effectiveness of surgical interventions including TKA. PROM scores act as a surrogate marker of real-world outcomes [19]. This is the largest reported single-surgeon series of TKA done using PSI with a minimum of 5 years follow-up. The retrospective cohort consists of an unselected group of consecutive patients operated under the care of the senior author. Our study has shown the importance of surgeon-directed planning to minimise the requirement of intra-operative changes and achieving an optimal outcome. The outcome scores were recorded in almost 90% of the patients who were alive with their primary implant at a mean follow-up period of 6.8 years. Multiple scoring systems were used to correlate functional outcomes and patient satisfaction. The concept of using the measured resection technique (to resurface the medial femoral condyle within the constraints of achieving neutral mechanical alignment) was also explored.

The OKS is widely used in both pre-and post-operative evaluation of outcomes in knee arthroplasty surgery [20]. It is also widely used by the national joint registries for monitoring and comparison [21]. Baker et al. [22] analysing PROM scores (collected between 6 to 12 months after TKA) in the National Joint Registry (NJR) reported mean pre-and post-operative OKS of 19.0 (SD 7.7) and 34.1 (SD 9.9), respectively. While the mean pre-operative OKS from our study was similar (19.2; SD 8.05), the mean post-operative score was better and appeared less variable (41.5; SD 7.95). Australian National Joint registry in their PROM pilot project reported that 44.3% of patients who underwent TKA post-operatively had an OKS score of 41 or above [19]. In comparison, 71.9% (*n* = 161) of patients in our study reported an OKS score of 41 or above at mid-term. The mean post-operative OKS in our study was also favourable to those reported by other authors for TKA performed using PSI (Table 7). Abane et al. [23] and Yan et al. [24] report in their randomised control trials (RCT) a mean OKS of 37.9 and 36.7, respectively for TKA performed using PSI.

**Table 7:** Demographics and planning details in RCTs that compared PSI and conventional TKA

Study	Exclusion criteria	Mean BMI	Total number of PSI knees (number of surgeons)	Follow up	Details of planning	Pre-op OKS
Abane et al. [23]	Extra-articular deformity, social circumstance impairing follow-up	28.8	59 (6)	3 months	Preliminary plan reviewed and approved. No details were given on planning changes. Correct size prediction 70%	NA
Yan et al. [24]	Previous knee operations, extra-articular deformity in tibia or femur, associated medical diseases affecting rehabilitation, non-compliant	NA	30 (3)	3 months	No details were given on pre-or intra-operative changes made	23.8 ± 5.6
Chen et al. [59]	Varus or valgus deformity > 11°, pre-operative fixed flexion deformity, 249/309 knees excluded	29.4	29 (1)	2 years	No changes were made either in the preliminary plan or intra-operatively	27 ± 10
Boonen et al. [60]	Non-compliant with post-operative rehabilitation protocol, or were unwilling to participate (342/522 excluded)	30.3	82 (3)	2 years	Preliminary plan checked before approval; no details of pre-or intra-operative changes	30.0 ± 9.3
Huijibregts et al. [61]	Previous osteotomy or fracture, non-correctable valgus deformity	NA	69 (2)	1 year	Plan assessed, no details of pre-operative changes. One change made intra-operatively	Median 20 (10)

^*^NA not available

The median post-operative OKS and gain in OKS reported by the NJR were 36 (IQR: 28–42) and 16 (IQR: 9–22) respectively [25]. Matharu et al.[26] also reported a median OKS gain of 16 for TKA. In comparison, the median post-operative OKS and gain in OKS in our study were 44 (IQR: 40–47) and 23 (IQR 16–30), respectively. Schoenmaker et al. [27] recently reported their outcomes on 177 TKA performed using the Signature system (Zimmer-Biomet Inc, Warsaw, IN, USA) with a 5-year follow-up. Their median OKS was 42 (IQR 33–46) although their median pre-operative score of 21 (IQR 16–26) was higher than in this cohort. The better patient pre-operative status in many of the RCTs is probably due to patient selection (Table 7)

Another parameter to assess patient satisfaction after TKA is the Forgotten Joint Score (FJS) which uses the patient's awareness of the artificial joint while performing the activities of daily living. We utilised the modified Forgotten Joint Score (MFJS) which is reported to be more reliable, better at assessing and discriminating high performing joint arthroplasties [11]. Both are scored between 0 and100, with a higher score indicating better outcome or ‘forgotten’ joint. Behrend et al. [28], the authors who validated the original FJS, reported a mean score of 86.6 and 79.3 in healthy male and female subjects respectively. An FJS score of more than 89 has been reported as a threshold for the perception of a prosthetic knee joint as a natural knee [29]. Although a similar comparative score for MFJS is not available, Robinson et al. [11] in their validation study for MFJS considered a score of 87.5 or more to be an ‘Excellent’ outcome. Carlson et al. [30] reported a mean FJS of 64.4 ± 29.0 at 5 years in a study cohort consisting of 566 TKA patients. They also reported that FJS is stable between 1- and 3-years post-operatively and decreases after 4 years. Other authors have reported a mean FJS between 47.0 and 60.6 in the post-operative period of less than 2 years [28–32]. Our mean MFJS score at a mean follow-up of 6.8 years was 72.86 ± 31.78.

The Beverland questionnaire is a simple 4-point tool to assess patient satisfaction after arthroplasty. Beverland [12] reported that while 81% of patients were 'Happy', only 4% were ‘Very happy’ at 10 years follow-up after total knee arthroplasty. A similar satisfaction scale adopted by the Australian Joint Registry (AJR) report 57.6% ‘Very Satisfied’ and 23.7% ‘Satisfied’ [19] corresponding to ‘Very Happy’ and ‘Happy’ respectively. In comparison, the proportion of patients in this cohort reporting ‘Very happy’ and ‘Happy’ were 75% and 11%, respectively. The proportion of dissatisfied patients in this cohort was only 4% (‘Never Happy’) which is lower than Beverland’s cohort (7%) and AJR (‘Dissatisfied’ and ‘Very Dissatisfied’ = 10%).

Five knees were revised. All were women, with a mean age of 68.4 years (range 57–78 years) and a mean BMI of 36.4 (range: 30.9–42). Three early failures occurred in the tibial side of the uncemented implants. This may be due to patient selection. Uncemented tibial component failures have been reported in the literature with high BMI as one of the contributing causes [33]. One patient with a cemented TKA had undergone revision at another centre for unexplained pain with little improvement. Another patient with cemented TKA presented with delayed patellar subluxation following a fall and subsequently required revision. Pre-operatively, she has been under the care of the stroke team with left parietal infarct, unsteady gait, and frequent falls. The revision rate of 1.6% (*n* = 5/298) is well within the 5-year implant survival of 93–97% which has been reported by others [34] [35].

Component malposition during TKA has been associated with poorer patient outcomes [36]. Studies have shown that a well-aligned prosthesis improves function and faster rehabilitation in patients who undergo TKA [5]. A recent meta-analysis by Gong S et al. [6] demonstrated that PSI can aid in improving femoral component alignment in comparison to conventional TKA. Mannan A et al. [37] also reported improvement in component alignment with PSI in their systemic review and meta-analysis.

Many of the RCTs and systemic reviews show similar functional outcomes between PSI and conventional instrumentation [7, 38]. However, the numbers are often small, learning curves may be included and there is usually very little detail on the surgeon-directed changes to the preliminary plan (Table 7). Boonen et al. [8, 27] presented their series of 200 consecutive TKA's performed using PSI with favourable results at 2 years and five-year follow-up. Dossett et al. [39] in their RCT report statistically significant improvement in functional outcome measures with PSI compared to conventional instrumentation.

Stronach et al. [40] reported inaccuracy of 3-D moulds (Signature system, Zimmer-Biomet, Warsaw, IN, USA) in predicting implant sizes and the need to make a significant number of intra-operative changes. However, very little information was provided on the surgeon-directed changes to the preliminary plan (in the legacy Signature system, surgeon approval was not obligatory; the 3-D moulds were automatically produced once the approval deadline passed which was usually 3 working days from when the preliminary plan was produced). In contrast, with careful pre-operative planning, we required only a minimal number of intra-operative changes. Pietsch et al.[41] and Boonen et al.[8] also noted the importance of surgeon-directed pre-operative planning in minimising intra-operative changes. The ability of different 3D planning software to enable careful pre-operative planning also may vary. Among 3-D planning software, the cartilage mapping visualisation was unique to ZPSIP^®^ software which was used in this study.

Both computed tomography (CT) and MRI scans can be used to plan and create PSI guides however studies have shown superiority of MRI over CT in relation to accuracy [42, 43]. Vincent et al. [42] in their meta-analysis compared accuracy of MRI based PSI to the CT based PSI and concluded that MRI based guides were able to demonstrate lower proportion of outliers. In a cadaver based study, Tibesku et al. [43] concluded that the CT based technique is inaccurate in presence of cartilage wear. Although, MRI scans are more expensive and time consuming than CT scan [44], they do avoid the risks from exposure to ionising radiation associated with CT scans. Pfitzner et al. [45] in their randomised clinical trial noted that operative time was shorter with PSI guides based on MRI scan in comparison to CT scan. Improved accuracy and safety associated with MRI scans were the reasons for our choice, which are adequately supported in the literature.

We planned the rotational alignment of the femoral component based on the surgical epicondylar axis and the tibial component at the junction of the medial third and lateral two-thirds of the tibial tuberosity. Rotational alignment in reference to the above landmarks have been shown to improve patello-femoral tracking [46–48].

Conventional instrumentations use a central pivot point to position the 4:1 cutting block in relative external rotation to the posterior condylar axis. This automatically results in the width of bone resection from the postero-medial condyle exceeding the implant thickness, resulting in decreased posterior condylar offset [49]. Excessive external rotation of the femoral component can cause flexion instability from an oversized medial flexion gap [50]. Restoring the posterior condylar offset has been proven to improve knee kinematics, range of movement and minimise flexion instability [49, 51, 52].

Kamenega et al. reported that the medial compartment stability in flexion and extension is associated with better functional outcomes [53]. The ZPSIP^®^ planning software allowed us to maintain the posterior condylar offset regardless of the external rotation chosen. This probably enabled us to achieve a stable medial compartment both in extension and flexion with 87% of this cohort reporting ‘very secure’ knees.

The mean post-operative pain scores after TKA using PSI reported by authors range from 0.8 to 1.4, 0 referring to no pain and 10 being the maximum possible pain experienced [54, 55]. The mean score reported in our study was 1.5 (SD 2.18), although this is at a minimum 5 years follow-up, whereas the above-mentioned authors report their findings at 3 months to less than 1-year follow-up. Australian joint registry data showed 63.1% of patients had a post-operative pain score of 2 or less on a scale of 0–10. In comparison, 77.8% (*n* = 173) of patients in our study had a score of 2 or less despite none having patellar resurfacing at the index surgery.

Biomechanical studies have shown increased contact forces affecting knee range of motion due to over-stuffing of the patello-femoral joint, and although clinical studies have not shown a clear correlation between over-stuffing the patello-femoral joint and poorer functional outcomes, many authors still advocate erring towards downsizing the femoral component and accepting minimal notching [56–58]. The pre-operative planning helped us to avoid the use of larger-sized femoral components by making minor adjustments in positioning the femoral component and accepting minimal notching in 193 cases (65%).

We identify some limitations of this study; there was no control group to compare the results of other techniques. Only a single PSI system was used, and this may not be representative of other systems. Objective evaluations (such as range of movement assessment, objective stability assessment, radiological assessment, or gait analysis) were also not available.

## Conclusion

Our study has shown that using PSI along with careful surgeon-directed pre-operative planning may minimise the need for intra-operative changes and deliver excellent functional outcomes and patient satisfaction. A majority of the patients who are essentially pain-free and perceived their knees to be stable, report ‘Very Happy’ in response to the satisfaction questionnaire. Our study also suggests that for the majority of patients to be ‘Very Happy’ a much greater post-operative OKS needs to be achieved than what has been suggested to be the expected mean post-operative OKS in the literature. Our experience, with the principles described above, demonstrate an approach and technique to deliver the desired outcomes.

## References

[1] Carr AJ, Robertsson O, Graves S (2012). Knee replacement. Lancet (London, England).

[2] Evans JT, Walker RW, Evans JP (2019). How long does a knee replacement last? A systematic review and meta-analysis of case series and national registry reports with more than 15 years of follow-up. Lancet.

[3] Beswick AD, Wylde V, Gooberman-Hill R (2012). What proportion of patients report long-term pain after total hip or knee replacement for osteoarthritis? A systematic review of prospective studies in unselected patients. BMJ Open.

[4] Jeffery RS, Morris RW, Denham RA (1991). Coronal alignment after total knee replacement. J Bone Joint Surg Br.

[5] Longstaff LM, Sloan K, Stamp N (2009). Good alignment after total knee arthroplasty leads to faster rehabilitation and better function. J Arthroplasty.

[6] Gong S, Xu W, Wang R (2019). Patient-specific instrumentation improved axial alignment of the femoral component, operative time and perioperative blood loss after total knee arthroplasty. Knee Surg Sports Traumatol Arthrosc.

[7] Mannan A, Akinyooye D, Hossain F (2017). A meta-analysis of functional outcomes in patient-specific instrumented knee arthroplasty. J Knee Surg.

[8] Boonen B, Schrander DE, Schotanus MGM, Hulsmans FJ, Kort NP (2015) Patient Specific Guides in total Knee Arthroplasty: a two year follow up of the first two hundred consecutive cases performed by a single Surgeon. JCRMM 1:1–10. https://www.researchgate.net/profile/Bert-Boonen/publication/291660250_Patient_Specific_Guides_in_Total_Knee_Arthroplasty_a_two_year_follow-up_of_the_first_two_hundred_consecutive_cases_of_a_single_surgeon/links/56a49ceb08ae1b65113255fb/Patient-Specific-Guides-in-Total-Knee-Arthroplasty-a-two-year-follow-up-of-the-first-two-hundred-consecutive-cases-of-a-single-surgeon.pdf. Accessed 12 Sept 2022

[9] León-Muñoz VJ, Martínez-Martínez F, López-López M, Santonja-Medina F (2019). Patient-specific instrumentation in total knee arthroplasty. Expert Rev Med Devices.

[10] Dawson J, Fitzpatrick R, Murray D, Carr A (1998). Questionnaire on the perceptions of patients about total knee replacement. J Bone Joint Surg Br.

[11] Robinson PG, Rankin CS, Lavery J (2018). The validity and reliability of the modified forgotten joint score. J Orthop.

[12] Beverland D (2010). Patient satisfaction following TKA: bless them all!. Orthopedics.

[13] Dunbar MJ, Robertsson O, Ryd L (2004). What’s all that noise? The effect of co-morbidity on health outcome questionnaire results after knee arthroplasty. Acta Orthop Scand.

[14] Cobb JP, Dixon H, Dandachli W, Iranpour F (2008). The anatomical tibial axis: Reliable rotational orientation in knee replacement. J Bone Jt Surg Ser B.

[15] Bellemans J, Vandenneucker H, Van Lauwe J, Victor J (2010). A new surgical technique for medial collateral ligament balancing Multiple needle puncturing. J Arthroplasty.

[16] Altay MA, Ertürk C, Altay N (2012). Patellar denervation in total knee arthroplasty without patellar resurfacing: a prospective, randomized controlled study. Orthop Traumatol Surg Res.

[17] Deekshith SRK, Reddy KJ, Raviteja R (2020). Patelloplasty in total knee arthroplasty with circumpatellar denervation versus without denervation—a randomized prospective study. Arthroplasty.

[18] Scott RD, Chmell MJ (2008). Balancing the posterior cruciate ligament during cruciate-retaining fixed and mobile-bearing total knee arthroplasty. description of the pull-out lift-off and slide-back tests. J Arthroplasty.

[19] Australia Orthopaedic Association National Joint Replacement Registry. AOA PROMs Pilot Project 2020. https://aoanjrr.sahmri.com/documents/10180/681914/aoanjrr+proms+pilot+fina531l+report. Accessed 19 Nov 2021

[20] Jenny JY, Diesinger Y (2012). The Oxford Knee Score: compared performance before and after knee replacement. Orthop Traumatol Surg Res.

[21] Rolfson O, Bohm E, Franklin P (2016). Patient-reported outcome measures in arthroplasty registries: report of the patient-reported outcome measures working group of the international society of arthroplasty registries part II. Recommendations for selection, administration, and analysis. Acta Orthop.

[22] Baker PN, Deehan DJ, Lees D (2012). The effect of surgical factors on early patient-reported outcome measures (PROMS) following total knee replacement. J Bone Jt Surg - Ser B.

[23] Abane L, Anract P, Boisgard S (2015). A comparison of patient-specific and conventional instrumentation for total knee arthroplasty: a multicentre randomised controlled trial. Bone Joint J.

[24] Yan CH, Chiu KY, Ng FY (2015). Comparison between patient-specific instruments and conventional instruments and computer navigation in total knee arthroplasty: a randomized controlled trial. Knee Surgery, Sport Traumatol Arthrosc.

[25] National Joint Registry for England, Wales and Northern Ireland. 10th Annual Report 2013. https://www.hqip.org.uk/wp-content/uploads/2018/02/national-joint-registry-10th-annual-report-2013.pdf. Accessed 12 Sept 2022

[26] Matharu GS, McBryde CW, Robb CA, Pynsent PB (2014). An analysis of Oxford hip and knee scores following primary hip and knee replacement performed at a specialist centre. Bone Jt J.

[27] Schoenmakers DAL, Schotanus MGM, Boonen B, Kort NP (2018). Consistency in patient-reported outcome measures after total knee arthroplasty using patient-specific instrumentation: a 5-year follow-up of 200 consecutive cases. Knee Surg Sports Traumatol Arthrosc.

[28] Behrend H, Giesinger K, Giesinger JM, Kuster MS (2012). The “Forgotten Joint” as the ultimate goal in joint arthroplasty. validation of a new patient-reported outcome measure. J Arthroplasty.

[29] Thijs E, Theeuwen DMJ, Boonen B (2014). Patient-specific instrumentation does not shorten surgical time: a prospective, randomized trial. Knee Surg Sports Traumatol Arthrosc.

[30] Carlson VR, Post ZD, Orozco FR (2018). When does the knee feel normal again: a cross-sectional study assessing the forgotten joint score in patients after total knee arthroplasty. J Arthroplasty.

[31] Kim KK, Howell SM, Won YY (2020). Kinematically aligned total knee arthroplasty with patient-specific instrument. Yonsei Med J.

[32] Lee QJ, Chang WYE, Wong YC (2020). Forgotten joint score for early outcome assessment after total knee arthroplasty: is it really useful?. Knee Surg Relat Res.

[33] Meneghini RM, de Beaubien BC (2013). Early failure of cementless porous tantalum monoblock tibial components. J Arthroplasty.

[34] Gøthesen O, Espehaug B, Havelin L (2013). Survival rates and causes of revision in cemented primary total knee replacement: a report from the Norwegian arthroplasty register 1994–2009. Bone Joint J.

[35] Khan M, Osman K, Green G, Haddad FS (2016). The epidemiology of failure in total knee arthroplasty: avoiding your next revision. Bone Joint J.

[36] Huang NFR, Dowsey MM, Ee E (2012). Coronal alignment correlates with outcome after total knee arthroplasty: 5-year follow-up of a randomized controlled trial. J Arthroplasty.

[37] Mannan A, Smith TO (2016). Favourable rotational alignment outcomes in PSI knee arthroplasty: a Level 1 systematic review and meta-analysis. Knee.

[38] Kizaki K, Shanmugaraj A, Yamashita F (2019). Total knee arthroplasty using patient-specific instrumentation for osteoarthritis of the knee: a meta-analysis. BMC Musculoskelet Disord.

[39] Dossett HG, Estrada NA, Swartz GJ (2014). A randomised controlled trial of kinematically and mechanically aligned total knee replacements. Bone Joint J.

[40] Stronach BM, Pelt CE, Erickson J, Peters CL (2013). Patient-specific total knee arthroplasty required frequent surgeon-directed changes. Clin Orthop Relat Res.

[41] Pietsch M, Djahani O, Hochegger M (2013). Patient-specific total knee arthroplasty: the importance of planning by the surgeon. Knee Surg Sports Traumatol Arthrosc.

[42] An VVG, Sivakumar BS, Phan K (2017). Accuracy of MRI-based vs. CT-based patient-specific instrumentation in total knee arthroplasty: a meta-analysis. J Orthop Sci.

[43] Tibesku CO, Innocenti B, Wong P (2012). Can CT-based patient-matched instrumentation achieve consistent rotational alignment in knee arthroplasty?. Arch Orthop Trauma Surg.

[44] Asada S, Mori S, Matsushita T (2014). Comparison of MRI and CT-based patient-specific guides for total knee arthroplasty. Knee.

[45] Pfitzner T, Abdel MP, von Roth P (2014). Small improvements in mechanical axis alignment achieved with MRI versus CT-based patient-specific instruments in TKA: a randomized clinical trial. Clin Orthop Relat Res.

[46] Poilvache PL, Insall JN, Scuderi GR, Font-Rodriguez DE (1996). Rotational landmarks and sizing of the distal femur in total knee arthroplasty. Clin Orthop Relat Res.

[47] Kim YH, Park JW, Kim JS, Park SD (2014). The relationship between the survival of total knee arthroplasty and postoperative coronal, sagittal and rotational alignment of knee prosthesis. Int Orthop.

[48] Nicoll D, Rowley DI (2010). Internal rotational error of the tibial component is a major cause of pain after total knee replacement. J Bone Jt Surg Ser B.

[49] Massin P, Gournay A (2006). Optimization of the posterior condylar offset, tibial slope, and condylar roll-back in total knee arthroplasty. J Arthroplasty.

[50] Olcott CW, Scott RD (2000). A comparison of 4 intraoperative methods to determine femoral component rotation during total knee arthroplasty. J Arthroplasty.

[51] Arabori M, Matsui N, Kuroda R (2008). Posterior condylar offset and flexion in posterior cruciate-retaining and posterior stabilized TKA. J Orthop Sci Off J Japanese Orthop Assoc.

[52] Seo S-S, Ha D-J, Kim C-W, Choi J-S (2009). Effect of posterior condylar offset on cruciate-retaining mobile TKA. Orthopedics.

[53] Kamenaga T, Muratsu H, Kanda Y (2018). The influence of postoperative knee stability on patient satisfaction in cruciate-retaining total knee arthroplasty. J Arthroplasty.

[54] Pietsch M, Djahani O, Hochegger M (2013). Patient-specific total knee arthroplasty: the importance of planning by the surgeon. Knee Surgery, Sport Traumatol Arthrosc.

[55] Vundelinckx BJ, Bruckers L, De Mulder K (2013). Functional and radiographic short-term outcome evaluation of the visionaire system, a patient-matched instrumentation system for total knee arthroplasty. J Arthroplasty.

[56] Beldman M, Breugem SJM, van Jonbergen H-PW (2015). Overstuffing in total knee replacement: no effect on clinical outcomes or anterior knee pain. Int Orthop.

[57] Pierson JL, Ritter MA, Keating EM (2007). The effect of stuffing the patellofemoral compartment on the outcome of total knee arthroplasty. J Bone Joint Surg Am.

[58] Matz J, Lanting BA, Howard JL (2019). Understanding the patellofemoral joint in total knee arthroplasty. Can J Surg.

[59] Chen JY, Chin PL, Tay DKJ (2015). Functional outcome and quality of life after patient-specific instrumentation in total knee arthroplasty. J Arthroplasty.

[60] Boonen B, Schotanus MGM, Kerens B (2016). No difference in clinical outcome between patient-matched positioning guides and conventional instrumented total knee arthroplasty 2 years post-operatively. Bone Joint J.

[61] Huijbregts HJTAM, Khan RJK, Fick DP (2016). Component alignment and clinical outcome following total knee arthroplasty: a randomised controlled trial comparing an intramedullary alignment system with patient-specific instrumentation. Bone Jt J.

